# TLR-3 is Present in Human Adipocytes, but Its Signalling is Not Required for Obesity-Induced Inflammation in Adipose Tissue *In Vivo*

**DOI:** 10.1371/journal.pone.0123152

**Published:** 2015-04-13

**Authors:** Dov B. Ballak, Edwin J. P. van Asseldonk, Janna A. van Diepen, Henry Jansen, Anneke Hijmans, Leo A. B. Joosten, Cees J. Tack, Mihai G. Netea, Rinke Stienstra

**Affiliations:** 1 Department of Internal Medicine, Radboud University Medical Center, Nijmegen, The Netherlands; 2 Department of Human Nutrition, Wageningen University and Research Centre, Wageningen, The Netherlands; University of Cambridge, UNITED KINGDOM

## Abstract

Innate immunity plays a pivotal role in obesity-induced low-grade inflammation originating from adipose tissue. Key receptors of the innate immune system including Toll-like receptors-2 and -4 (TLRs) are triggered by nutrient excess to promote inflammation. The role of other TLRs in this process is largely unknown. In addition to double-stranded viral mRNA, TLR-3 can also recognize mRNA from dying endogenous cells, a process that is frequently observed within obese adipose tissue. Here, we identified profound expression of TLR-3 in adipocytes and investigated its role during diet-induced obesity. Human adipose tissue biopsies (n=80) and an adipocyte cell-line were used to study TLR-3 expression and function. TLR-3-/- and WT animals were exposed to a high-fat diet (HFD) for 16 weeks to induce obesity. Expression of TLR-3 was significantly higher in human adipocytes compared to the non-adipocyte cells part of the adipose tissue. *In vitro*, TLR-3 expression was induced during differentiation of adipocytes and stimulation of the receptor led to elevated expression of pro-inflammatory cytokines. *In vivo*, TLR-3 deficiency did not significantly influence HFD-induced obesity, insulin sensitivity or inflammation. In humans, TLR-3 expression in adipose tissue did not correlate with BMI or insulin sensitivity (HOMA-IR). Together, our results demonstrate that TLR-3 is highly expressed in adipocytes and functionally active. However, TLR-3 appears to play a redundant role in obesity-induced inflammation and insulin resistance.

## Introduction

Obesity is a worldwide problem that profoundly affects global health [1]. Obesity is associated with type 2 diabetes mellitus, cardiovascular disease, hepatic steatosis and obesity-related cancers [2]. Development of obesity leads to morphological and functional changes in adipose tissue. Adipocytes become hypertrophic and immune cells, such as macrophages [3,4], T- and B-cells [5], infiltrate into the adipose tissue [6]. This inflammatory trait is associated with insulin resistance and subsequently type 2 diabetes mellitus. Much remains to be learned about the factors that initiate and propagate adipose tissue inflammation.

An inflammatory response is initiated when exogenous (pathogen associated molecular pattern, PAMP) or endogenous (danger associated molecular pattern, DAMP) ligands are recognized by a pattern recognition receptor (PRR). Activation of PRRs triggers intracellular signalling cascades, eventually leading to production of chemokines and pro-inflammatory cytokines and recruitment and activation of immune cells. TNFα, interleukin (IL)-1β, IL-6 and IL-8 are the best-known cytokines involved in adipose tissue inflammation and the subsequent development of insulin resistance [7–9]. Potential DAMPs in adipose tissue, released by dying adipocytes or certain lipids, are present in increased concentrations in obesity [10]. It is therefore of high interest to identify which pattern recognition receptors mediate the link between obesity and adipose tissue inflammation.

Toll-like receptors (TLRs) are a large class of transmembrane PPRs and well known for their role in inflammatory signalling during infection and inflammation [11]. In humans, ten members of the TLR-family have been described [12]. Amongst these, TLR-2 and TLR-4 have emerged as important regulators of pro-inflammatory signalling in response to saturated fatty acids [13–16], thereby mediating at least part of the HFD-induced adipose tissue inflammation [17]. In contrast, the role of other TLRs in adipose tissue inflammation remains largely unknown.

In the present study we screened the expression of all TLR-family members in adipose tissue. Remarkably, we identified TLR-3 as being highly expressed in the adipocytes, in contrast to other TLRs that were mainly present in the stromal vascular fraction of the adipose tissue. Interestingly, TLR-3 can bind mRNA that is released from dying cells [18] aside from double-stranded viral mRNA [19], hence may be activated by apoptotic adipocytes that are frequently observed within obese adipose tissue [20]. Therefore, we were prompted to investigate the role of TLR-3 in adipose tissue inflammation, using complementary *in-vitro* and *in-vivo* experimental models, as well as human adipose tissue biopsies.

## Methods

### Human subjects

Paired subcutaneous (SAT) and visceral adipose tissue (omentum) (VAT) samples were obtained according a standardized procedure from 4 patients (two females and two males) to investigate expression of TLRs in mature adipocytes (MA) versus the stromal vascular fraction (SVF). Tissue biopsies were taken during elective cholecystectomy. Subjects were between 40–60 years old with a body mass index (BMI) of 25–28 kg/m^2^. Subjects were normoglycemic. Metabolic, endocrine and chronic and/or acute inflammatory diseases were excluded.

Moreover, adipose tissue samples were obtained from 80 healthy human subjects who were recruited through advertisements in local newspapers. We included healthy subjects between 40 and 70 years old (see S1 Table). Subcutaneous adipose tissue biopsies were obtained under local anesthesia by needle biopsies 6–10 cm lateral to the umbilicus. Samples were taken after an overnight fast. TLR-3 expression was measured in the adipose tissue biopsies of all subjects. Subsequently, metabolic parameters were compared to TLR-3 expression: BMI (<25, n = 33; BMI>30, n = 18), Homeostatic model assessment for insulin resistance (HOMA-IR) levels (<2, n = 40; >2 n = 26, hsCRP plasma levels (<0.5mg/l, n = 19; >1.7mg/l, n = 19) and number of crown-like structures in the adipose tissue (no, n = 53; yes, n = 19). Furthermore, associations were made between adipose tissue TLR-3 expression and the lowest and highest quartiles, with respect to mRNA expression levels, of IL-8, monocyte chemoattractant protein (MCP)-1, fatty acid binding protein (FABP)4 and adipocyte size in the adipose tissue. These are markers for either adipose tissue inflammation or adipocyte health. The tissue samples were collected after written informed consent from all individual participants, and the protocol was approved by the ethical committee of the Radboud University Nijmegen Medical Centre and in accordance with the Declaration of Helsinki.

### Stromal vascular and mature adipocyte fractions

The collected SAT and VAT samples were disaggregated using collagenase type 1 (Gibco, Life Technologies) digestion to isolate adipocytes and the stromal vascular fraction (SVF) as described before [21]. Purity of the two different fractions was confirmed using FABP 4 (adipocytes), and CD45 (hematopoietic cells) (S1 Table). The cellular fractions were subsequently used for RNA isolation, followed by real-time PCR analysis, as described below.

### Histochemistry

Morphometry of individual fat cells was assessed using digital image analyses as described previously [22]. In short: for each subject, the adipocyte cell diameter of all fat cells in five to ten microscopic fields of view were counted and measured. For detection of crown-like structures, a CD68 antibody (AbD Serotec, Oxford, UK) was used in human samples. In mouse samples, an antibody against F4/80^+^ (AbD Serotec, Oxford, UK) was used. Visualization of the complex was done using 3,3’-diaminobenzidene for 5 min. Negative controls were used by omitting the primary antibody.

### Animals

16 Male TLR-3-/- and 20 male WT mice on a C57Bl/6 background were obtained from Jacksons Laboratory and housed in a pathogen-free environment in the animal facility of the Radboud University, Nijmegen. The mice were purchased at Jackson and housed individually in filter top cages in a separate room, with water and food *ad libitum*. The housing temperature was held at 23°C and a 12:12h light-dark cycle was maintained. After a 1-week run-in period on a low-fat diet, the mice were divided in a low-fat diet (LFD) and a high-fat diet (HFD) group. The diet contained either 10% or 45% energy derived from fat, lard-oil was replaced by palm-oil (D12450B or 12451; Research Diet Services, Wijk bij Duurstede, The Netherlands). This diet was continued for 16 weeks. Bodyweight of the animals was recorded weekly. After sacrifice, liver and epididymal adipose tissue weight were measured. All animal procedures were conducted under protocols approved by the animal experimentation committee of Radboud University Medical Centre, Nijmegen.

### Insulin tolerance test (ITT)

An insulin tolerance test was performed after a 4 hours fasting period. 0,75 U of insulin per kilogram bodyweight was injected intraperitoneally. Blood glucose levels were determined with an Accu-chek glucosemeter (Roche Diagnostics, Almere, The Netherlands) at indicated time points after insulin administration.

### qPCR

Total RNA was isolated from adipose tissue using TRIzol (Invitrogen, Carlsbad, CA), according to manufacturer’s instructions. RNA was reverse-transcribed (iScript cDNA Synthesis Kit, Bio-Rad Laboratories). RT-PCR was performed using specific primers (see S2 Table), power SYBR green master mix (Applied Biosystems, Foster City, CA) using the Step-one Real-Time PCR system (Applied Biosystems, Foster City, CA). For mice samples, we used 36B4 as housekeeping gene. For human samples we used B2M as a housekeeping gene.

### Insulin

Insulin levels in humans were measured by the clinical laboratory unit of the Radboud University Medical Centre, Nijmegen.

### Plasma glucose

Glucose (Liquicolor, Human GmbH, Wiesbaden, Germany) was measured enzymatically following manufacturers’ protocols.

### Cell lines

Human Simpson-Golabi-Behmel syndrome (SGBS) preadipocytes were cultured as described earlier [23]. After full differentiation cells were treated with poly(I:C) 12.5 μg/ml or lipopolysaccharide 50 ng/ml to stimulate respectively TLR-3 or TLR-4.

### Western Blot

Human subcutaneous adipose tissue samples were analysed for TLR-3 expression. Lysates prepared using a lysis buffer [50mM Tris (pH7.4), 150mMNaCl, 2mMEDTA,1%Nonidet P-40, 50mMNaF,and 0.25% sodium deoxycholate with phosstop phosphatase-inhibitor cocktail tablet (Roche) and complete, EDTA-free protease-inhibitor cocktail tablet (Roche). The homogenate was centrifuged at 4°C for 10 min at 18.000 rcf and the supernatant was used for Western blot analysis. Equal amounts of protein, as determined by a BCA protein assay (Thermo FisherScientific, Rockford, IL) were separated using a polyacrylamide SDS page gel. After SDS-PAGE, proteins were transferred to a nitrocellulose membrane using a Trans-Blot Turbo Transfer System (Biorad) following manufacturer’s instructions. The membrane was blocked with 5% (wt/vol) milk powder in Tris-buffered saline (TBS)/Tween 20 for 1 h at room temperature followed by incubation overnight at 4 C with a TLR-3 antibody (Abcam, ab62566), in 5% (wt/vol) milk powder/TBS/Tween 20. After overnight incubation, the blots were incubated with horseradish peroxidase-conjugated secondary antibody (goat anti-rabbit, A0545, Sigma Aldrich) at a dilution of 1:5000 in 5% (wt/vol) milk powder in TBS/Tween 20 for 1 h at room temperature and subsequently developed with ECL plus (Thermo Scientific) according to the manufacturer’s instructions. Bands were visualized using a ChemiDoc System (Biorad) and quantified using Image lab software (Biorad).

### Small interfering RNA

To specifically suppress TLR-3 expression in differentiated adipocytes, cells were transfected (X-tremeGENE siRNA Transfection Reagent, Roche) with small interfering (si)RNA against TLR-3 (Thermo Scientific). As a nonspecific control, scrambled siRNA (Thermo Scientific) was used. After 72 hours of incubation, gene expression was determined.

### Statistical analysis

Data are shown as means ± SEM. Differences between groups were analyzed using Student’s *t* test, differences among 4 groups were analyzed with ANOVA followed by *post-hoc* Bonferroni tests and correlations were evaluated with regression analysis in Graphpad Prism 6.0. p-values<0.05 were considered significant.

## Results

### TLR-3 is highly expressed in adipocytes and functionally active

**Fig 1:**
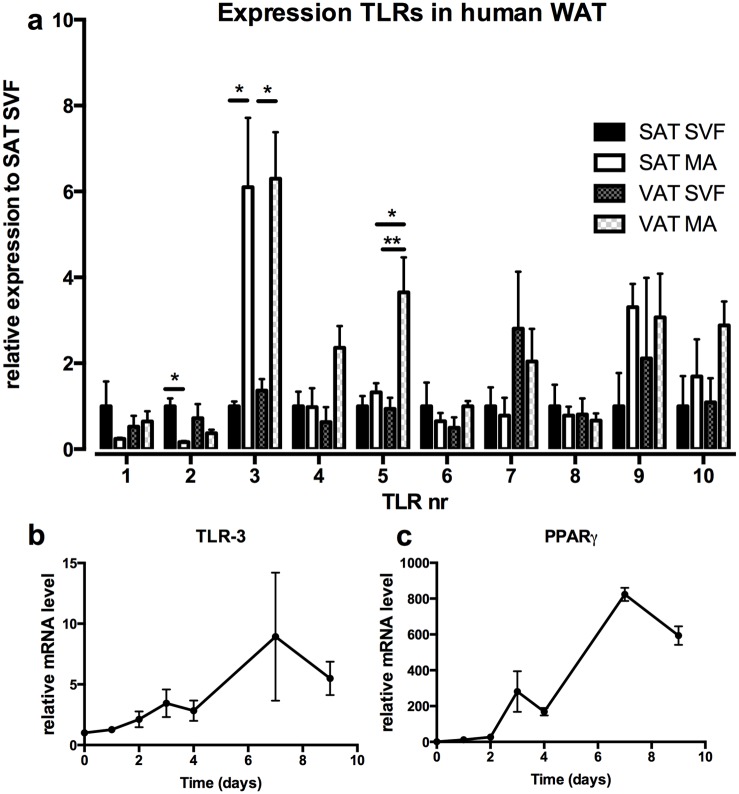
TLR-3 is predominantly expressed in adipocytes. (a) Biopsies from visceral- (VAT) and subcutaneous adipose tissue (SAT) were obtained from 4 healthy subjects and TLR expression was determined in stromal vascular fraction (SVF) and mature adipocytes (MA). mRNA levels of (b) TLR-3 and (c) PPAR-γ were measured during differentiation of human SGBS adipocytes. * p<0.05, ** p<0.01. Data are shown as means ± SEM.

We investigated expression patterns of all TLR-family members in human adipose tissue. Adipose tissue biopsies of both subcutaneous and visceral depots were used and separated in stromal vascular fraction (SVF) and mature adipocytes (MA). Separation of adipocyte and stromal vascular fraction was adequate as determined by expression of CD45 and FABP4 in the two fractions (S1a/S1b Fig). Most TLR-family members were more robustly expressed in the SVF fraction compared to the MA fraction (*e*.*g*. TLR-1, TLR-2), or similarly expressed between the SVF and MA fraction (*e*.*g*. TLR-6, TLR-7, TLR-8). Remarkably, the expression profile of TLR-3 was different from the expression pattern observed for the other TLRs, as it was significantly higher expressed in the MA fraction compared to SVF (Fig 1a). The only additional receptor that was relatively high expressed in adipocytes was TLR-5. However, its expression was just 4 times higher in only the visceral adipocytes compared to the stromal vascular cells, where TLR-3 had a 6-fold higher expression in adipocytes. This difference was apparent for both subcutaneous and visceral adipose tissue. Moreover, TLR-3 protein expression in human subcutaneous adipose tissue was confirmed by Western Blot (S1c Fig). Additionally, we found that TLR-3 was highly induced during differentiation of SGBS adipocytes *in vitro* (Fig 1b), in a similar fashion as PPAR-γ, a marker for adipocyte differentiation (Fig 1c).

**Fig 2:**
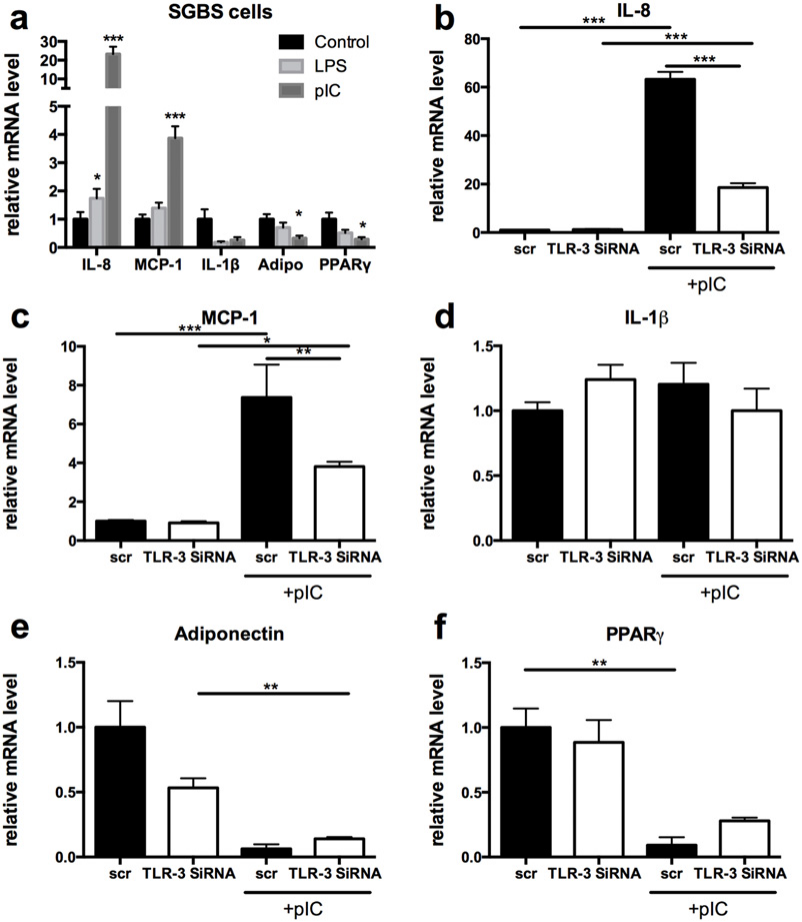
TLR-3 is functionally active in adipocytes. (a) Differentiated SGBS adipocytes were stimulated with either a TLR-3 (poly:IC 12.5μg/ml) or TLR-4 (LPS 50ng/ml) agonist. mRNA levels were measured for IL-8, MCP-1, IL-1 β, adiponectin and PPAR-γ. (b-f) SGBS adipocytes were treated with SiRNA against TLR-3 or scr SiRNA and stimulated with poly:IC. mRNA levels of (b) IL-8, (c) MCP-1, (d) IL-1β, (e) adiponectin, (f) PPAR- γ were subsequently measured. * p<0.05, ** p<0.01, *** p<0.001. Data are shown as means ± SEM.

Next, we tested whether TLR-3 activation in adipocytes was functionally active. Therefore, we treated differentiated adipocytes with poly(I:C), a synthetic TLR-3 ligand and compared this to E.coli LPS, a TLR-4 ligand. Similar to LPS, poly(I:C) significantly increased transcription of pro-inflammatory cytokine IL-8 after 24 hours. In addition, poly(I:C) induced expression of MCP-1, a key molecule that mediates macrophage infiltration in adipose tissue [24], while IL-1β expression was not increased after 24 hours of LPS or poly(I:C) treatment. Furthermore, adipogenic genes adiponectin and PPARγ were significantly decreased after Poly(I:C) stimulation (Fig 2a).

To ensure that the effects of poly(I:C) on adipogenic and pro-inflammatory cytokine gene expression were specifically mediated by TLR-3, the expression of TLR-3 was blocked using small interfering RNA (siRNA). TLR-3 was blocked with a knockdown of almost 75% (S2 Fig). These data reveal that poly(I:C) increased expression of IL-8 and MCP-1 in a TLR-3 dependent manner, since blockage of TLR-3 with siRNA greatly inhibited the poly(I:C) mediated induction of these genes (Fig 2b and 2c). Gene expression levels of IL-1β were unaffected (Fig 2d). In contrast, downregulation of adiponectin and PPARγ was not affected by blockage of TLR-3, suggesting that other signalling routes besides TLR-3 are likely to play a role in down-regulating adipogenesis upon Poly(I:C) stimulation (Fig 2e and 2f).

### TLR-3 deficiency does not affect weight gain or insulin sensitivity after HFD-feeding

**Fig 3:**
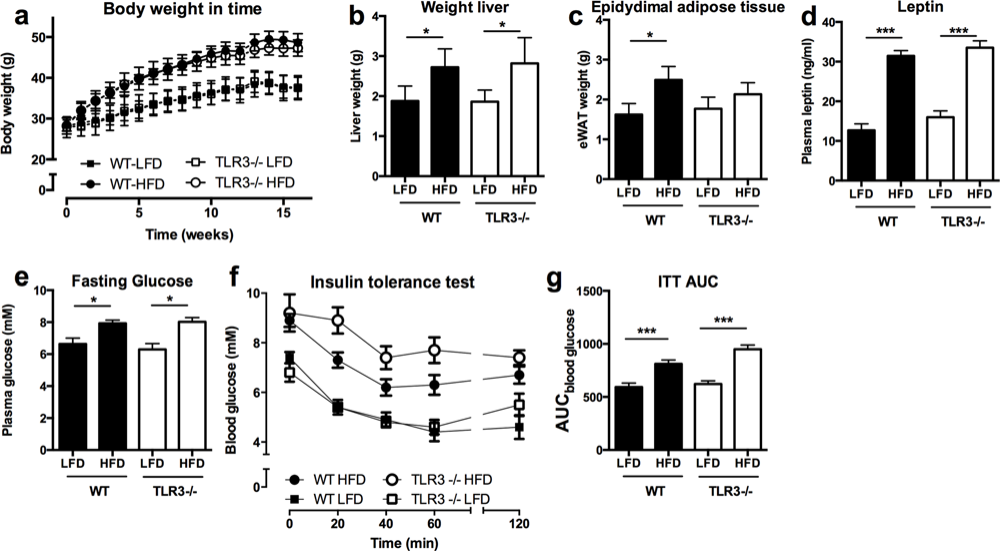
TLR-3 deficiency does not protect mice against metabolic abnormalities. Wild-type (WT) and TLR-3-/- mice were subjected to 16 weeks of low fat diet (LFD) or high fat diet (HFD). (a) development of the bodyweight, (b) liver weight, (c) epididymal adipose tissue weight, (d) plasma leptin levels, (e) fasting glucose levels, (f) insulin tolerance test (ITT), (g) area under the curve for ITT. * p<0.05, *** p<0.001. Number of mice per group: WT-LFD n = 10; WT-HFD n = 10; TLR-3-/-LFD n = 7; TLR-3-/-HFD n = 9. Data are shown as means ± SEM.

To test whether TLR-3 is functionally relevant in the process of diet induced obesity and insulin resistance, TLR-3-/- mice and WT controls were fed a HFD for 16 weeks. TLR-3 deficiency did not influence HFD-induced obesity, compared to WT mice (Fig 3a). Similarly, liver and epididymal adipose (eWAT) tissue weights were not statistically different in TLR-3-/-mice as compared to WT animals (Fig 3b and 3c). The latter was confirmed by a similar increase of plasma leptin levels in both genotypes due to HFD feeding (Fig 3d). Next, we tested whether TLR-3-/- mice were protected from HFD-induced insulin resistance. Fasting glucose levels (8 hours) were similar in both genotypes, after LFD and HFD-feeding (Fig 3e). An insulin tolerance test performed 15 weeks after starting the diet intervention showed a tendency towards a reduction in insulin sensitivity in HFD-fed TLR-3-/- mice, however this difference did not reach statistical (Fig 3f and 3g).

### TLR-3 deficiency does not influence obesity-induced adipose tissue inflammation in mice

**Fig 4:**
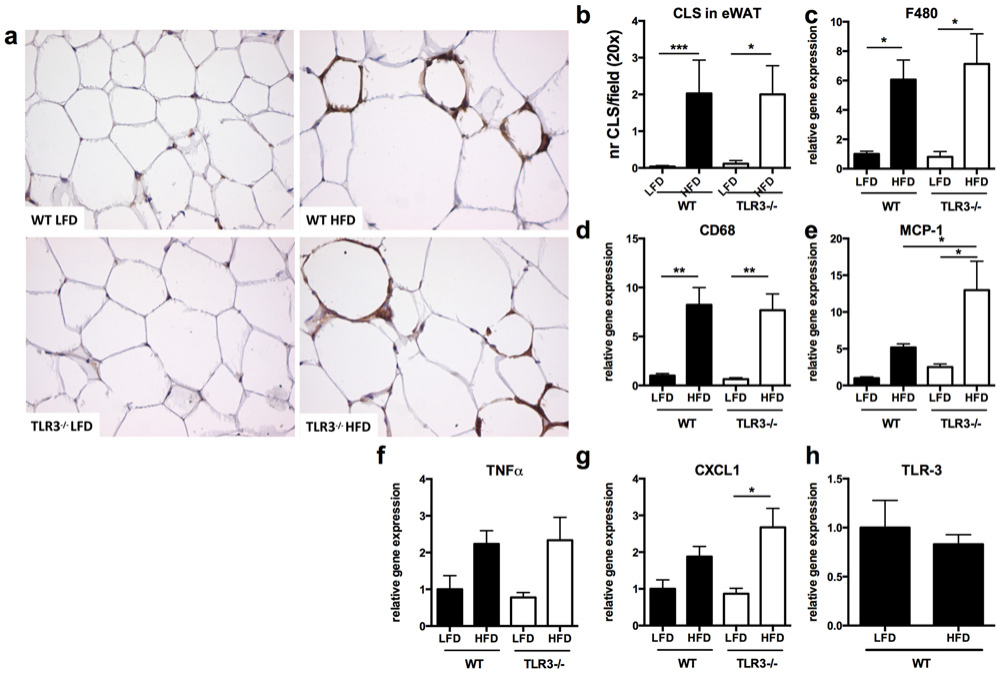
TLR-3 deficiency does not ameliorate adipose tissue inflammation. After 16 weeks of low fat diet (LFD) or high fat diet (HFD) intervention, adipose tissue of wild-type (WT) and TLR-3-/- mice was investigated for inflammatory parameters. (a) Adipose tissue of mice stained for F4/80, magnification 20x, (b) number of crown-like structures in adipose tissue. Inflammatory markers were measured (c) F480, (d) CD68, (e) MCP-1, (f) TNFα, (g) CXCL1. (h) mRNA levels of TLR-3 in WT mice fed a LFD or HFD for 16 weeks. * p<0.05, ** p<0.01. Number of mice per group: WT-LFD n = 10; WT-HFD n = 10; TLR-3-/-LFD n = 7; TLR-3-/-HFD n = 9. Data are shown as means ± SEM.

In a following set of experiments, we assessed whether TLR-3 deficiency affected the development of obesity-induced adipose tissue inflammation. Therefore, histological sections of eWAT were stained with F4-80, a macrophage marker (Fig 4a). No differences were found in the number of crown-like structures between TLR-3-/- and WT animals (Fig 4b). This result was confirmed by similar gene expression levels of two macrophage markers, F480 and CD68, as detected by qPCR analysis (Fig 4c and 4d). Only monocyte chemoattractant protein (MCP)-1 was slightly higher expressed in the absence of TLR-3 (Fig 4e). Together, these data suggest that TLR-3 deficiency does not affect macrophage infiltration. Additionally, we did not find differences in macrophage activation as determined by expression of the pro-inflammatory cytokines TNFα and CXCL1 in adipose tissue of TLR-3-/- and WT mice, (Fig 4f and 4g). Interestingly, TLR-3 expression itself was not different between HFD- and LFD-treated WT mice (Fig 4h), while expression of other TLRs was upregulated (TLR-6) or tended to increase (TLR-1 and TLR-2) in response to HFD, except for TLR-4 (S3a–S3d Fig). However, expression of these TLRs was not different between TLR-3-/- and WT mice fed either a LFD or a HFD, revealing no compensatory up-regulation of other TLRs in adipose tissue in the absence of TLR-3. Furthermore, expression of 9 TLRs in chow fed mice was measured in stromal vascular cells compared to mature adipocytes (S4 Fig). Most TLRs, except 2, 4 and 5 were more expressed in stromal vascular cells. Thus TLR-3, opposed to human adipose tissue, displayed a lower expression level in mouse adipocytes.

### TLR-3 in human subcutaneous adipose tissue

**Fig 5:**
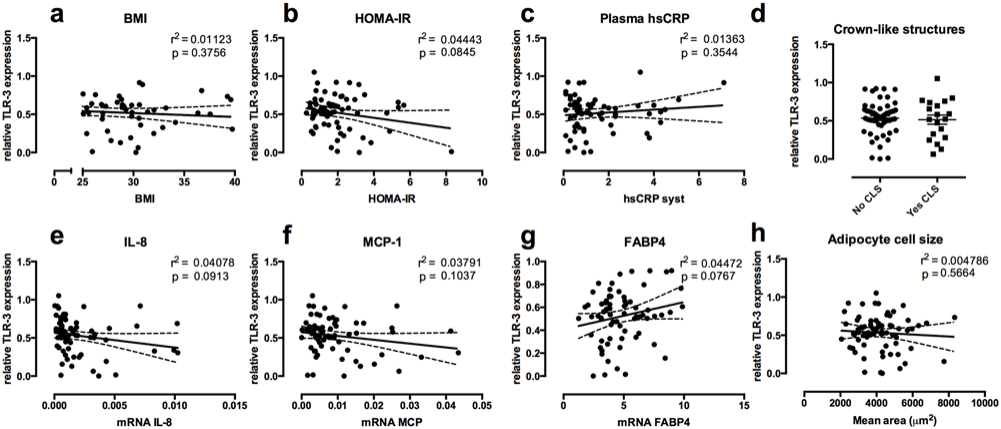
TLR-3 in human adipose tissue. Subcutaneous adipose tissue samples of 80 healthy individuals were obtained. TLR-3 mRNA levels were associated with (a) BMI, (b) HOMA-IR, (c) plasma CRP-levels, (d) number of crown-like structures in adipose tissue. Association of MAP3K8 mRNA expression in human subcutaneous adipose tissue with mRNA expression of (e) IL-8, (f) MCP-1, (g) FABP4 and (h) adipocytes cell size. * p<0.05. Data are shown as means ± SEM. HOMA-IR = Homeostatic Model Assessment for insulin resistance.

Next, we correlated the gene expression levels of TLR-3 in subcutaneous adipose tissue samples from 80 healthy donors, with markers of obesity, inflammation and insulin sensitivity. TLR-3 expression in subcutaneous adipose tissue was similar between lean and obese individuals (Fig 5a). Similarly, no difference in TLR-3 expression was observed between subjects with high insulin sensitivity levels versus low insulin sensitivity levels, as determined by the homeostatic model assessment for insulin resistance (HOMA-IR) (Fig 5b). Subjects with higher systemic inflammation (high sensitive C-reactive protein (hs-CRP) levels) or increased adipose tissue inflammation (number of crown-like structures) also showed an equal expression of TLR-3 (Fig 5c and 5d). In contrast, we found that adipose tissue of subjects with higher expression of the pro-inflammatory cytokine IL-8 showed a tendency for lower mRNA levels of TLR-3 (Fig 5e). A similar tendency was seen for MCP-1 and TLR-3 expression (Fig 5f). On the contrary, FABP4 showed a positive association with TLR-3 expression in adipose tissue (Fig 5g), although this did not reach statistical significance. Finally, despite a high expression of TLR-3 in mature adipocytes, its expression did not associate with mean adipocyte cell size in adipose tissue (Fig 5h).

## Discussion

Innate pattern recognition receptors (PRRs) are increasingly recognized as direct or indirect sensors of excessive nutrients instigating a chronic inflammatory response in adipose tissue during obesity, with possibly detrimental effects on insulin sensitivity levels [10]. During this metaflammation [7], the function of TLR-3, stimulated by double-stranded mRNA and yielding production of type-1 Interferons [19], remains largely unknown. In the current study we examined whether TLR-3-signalling is involved in the development of adipose tissue inflammation and insulin resistance during the development of obesity. To study a potential role of TLR-3 triggered by its remarkable high expression in adipocytes, as compared to other members of the TLR family. We show that TLR-3 is active in adipocytes upon stimulation with specific ligands. However, TLR-3-/- mice showed no significant amelioration in adipose tissue inflammation and insulin sensitivity after HFD-feeding. Additionally, data obtained from 80 human adipose tissue biopsies showed a limited association of TLR-3 gene expression levels with various metabolic abnormalities including insulin resistance and adipose tissue inflammation. Together, these data suggest that although TLR-3 is predominantly expressed in adipocytes and functionally active, it has a limited role in mediating 16 weeks HFD-induced adipose tissue inflammation and systemic insulin sensitivity in mice, nor a profound association with metabolic abnormalities in humans.

Interestingly, TLR-3 is preferentially expressed on adipocytes as compared to stromal vascular cells suggestive of a functional role of the receptor specifically in these cells. Indeed, our data demonstrate that stimulation of TLR-3 increases pro-inflammatory cytokine expression, an effect that was abolished by silencing of TLR-3 in adipocytes using siRNA. Thereby, we confirm that adipocytes can produce cytokines after specific TLR-3 stimulation [25]. Moreover, TLR-3 was up-regulated during differentiation of adipocytes. The high expression levels led us to believe that TLR-3 may exert a more dominant role in adipose tissue as compared to other TLR-family members.

However, despite its prominent expression and function in adipocytes, in vivo studies using TLR-3-/- mice revealed no amelioration in adipose tissue inflammation or insulin sensitivity levels as compared to WT mice after 16 weeks of HFD-feeding. The difference in expression pattern of TLR-3 in adipocytes vs stromal vascular fraction between mouse and human adipose tissue may explain at least some of our apparent conflicting observations between *in vitro* and *in vivo* results. In addition, we observed a different effect of TLR-3 deficiency on MCP-1 expression between the *in vitro* and *in vivo* experiments. These divergent responses may be explained by differences in model-complexity or a different response to TLR-3 signalling in human versus murine cells [26].

Our *in vivo* results are partly in contrast to a recent report demonstrating that the absence of TLR-3 or its blockage using a specific antibody led to improvements in glucose tolerance [27]. However, in line with our results, a glucose tolerance test performed by Wu *et al* after 14 weeks of HFD revealed no differences between WT and TLR-3-/- animals compared to WT mice. Only after 26 weeks of HFD-feeding, the absence of TLR-3 led to an improvement in glucose tolerance. Moreover, the absence of TLR-3 protected against the development of hepatic steatosis after 33 weeks of HFD and reduced expression levels of TNFα in liver. Our research advances these studies by specifically focussing on establishing a role for TLR-3 in adipose tissue. Possibly, TLR-3 exerts differential effects in the liver versus adipose tissue during the development of HFD-induced obesity. More likely, the opposite outcomes are explained by differences in study design. The diet intervention in our study was done using a low fat diet and high fat diet containing 45% of energy derived from fat. Wu *et al* used a high fat diet with 60% of energy from fat with chow diet as control feeding. Additionally, the intervention period lastly for a total period of 33 weeks whereas our study design included only 16 weeks of HFD-feeding.

Hence, these outcomes may suggest that the absence of TLR-3 ameliorates metabolic abnormalities induced by high fat diet feeding only after a prolonged time of diet intervention. One might speculate that the ligand responsible for activation of TLR-3 only becomes available after long term and very high fat diet feeding.

Although many TLRs have both endogenous and exogenous stimuli, the endogenous activator of TLR-3 remains to be established. Based on current knowledge, TLR-3 primary function is to recognize double stranded RNA and contributes to the host defence against viruses. What type of endogenous activator in adipose tissue might propel TLR-3 activation is currently unknown, however it has been shown that TLR-3 also recognizes single-stranded mRNA [18]. In obesity, dying adipocytes may therefore release mRNA, which is then recognized by TLR-3. However, in spite of its high expression levels in human adipocytes that may suggest otherwise, our results do not provide evidence for a vital, important role of TLR-3 in adipose tissue during diet-induced obesity. The high expression of TLR3 in the adipocyte fraction of human adipose tissue, however, suggest a possible other role in human adipocyte function. Recently, it was discovered that activation of TLR-3 reduces adipocyte differentiation [28] and that this resulted in inhibited insulin and glucose signalling [29]. As TLR-3 may be involved in the recognition of dying adipocytes, the necrotic cells may thereby directly influence surrounding pre-adipocytes and inhibit their differentiation and suppress glucose and insulin signalling. Moreover, although especially TLR-2 and TLR-4 have been implicated in mediating the pro-inflammatory actions of free fatty acids, we cannot rule out that TLR-3 mediates some of these free fatty acids related effects.

As it is hypothesised that viruses play a role in the pathogenesis of type 1 diabetes, it might be interesting to investigate the role of TLR-3 in the pancreas. Several studies have showed a link between TLR-3 induced islet inflammation and type 1 diabetes (22–24). Because 16 weeks is not long enough to induce islet inflammation in mice in contrary to adipose tissue inflammation, our study was able to investigate the effects of TLR-3 without confounding effects of the pancreas.

In line with the results from the animal study that did not reveal a vital role for TLR-3 in mediating obesity-induced inflammation and the development of insulin resistance, the function of the receptor in human adipose tissue appears to be relatively small. However, as shown in literature [27], TLR-3 may exacerbate metabolic abnormalities in the liver after 33 weeks of HFD in mice as opposed to adipose tissue. Moreover, we observed no clear associations in humans, suggesting that TLR-3 has a limited role in mediating adipose tissue inflammation and the development of insulin resistance. However, these results do not completely rule out any function of TLR-3-signalling in adipose tissue during the presence of obesity. Noticeably, our results were obtained using subcutaneous adipose tissue biopsies and not the metabolically more active visceral depot. In addition to gene expression levels, future studies should also determine the activity of TLR-3-dependent signalling routes within adipose tissue by measuring TLR-3 protein levels and possible intracellular downstream targets.

Altogether, these data show that TLR-3 is highly expressed in adipocytes as compared to the stromal vascular cells and is functionally active. Nevertheless, in mice after 16 weeks of HFD and in humans, the data does not support a fundamental role for TLR-3 in the development of obesity-induced adipose tissue inflammation and insulin resistance.

## Supporting Information

S1 Fig Human adipose tissue. Biopsies from visceral- (VAT) and subcutaneous adipose tissue (SAT) were obtained from 4 healthy subjects and TLR expression was determined in stromal vascular fraction (SVF) and mature adipocytes (MA). mRNA levels of (a) FABP4 (b) CD45. (c) Western blot was used to confirm TLR-3 protein expression in human subcutaneous adipose tissue. * p<0.05, ** p<0.01, *** p<0.001. Data are shown as means ± SEM.
(TIFF)

S2 Fig Knockdown of TLR-3 in SGBS cells. SGBS cells where transfected with small interference RNA against TLR-3 to reduce expression of TLR-3. Gene expression was determined after 72 hours. *** p<0.001. Data are shown as means ± SEM.
(TIFF)

S3 Fig TLR expression in epididymal adipose tissue after HFD. After 16 weeks of low fat diet (LFD) or high fat diet (HFD) intervention, adipose tissue of wild-type (WT) and TLR-3-/- mice was investigated for TLR expression. mRNA levels of (a) TLR-1, (b) TLR-2, (c) TLR-4 and (d) TLR-6 were measured. * p<0.05, ** p<0.01. Number of mice per group: WT-LFD n = 10; WT-HFD n = 10; TLR-3-/-LFD n = 7; TLR-3-/-HFD n = 9. Data are shown as means ± SEM.
(TIFF)

S4 Fig TLR expression in epididymal adipose tissue in chow-fed mice. mRNA levels of 9 different TLRs were measured adipocytes versus stromal vascular cells in chow fed mice. Number of mice per group: adipocytes n = 9, stromal vascular cells n = 9. Data are shown as means ± SEM. * p<0.05, ** p<0.01, *** p<0.001. Data are shown as means ± SEM.
(TIFF)

S1 Table Subject characteristics. This table lists information on the subjects of which adipose tissue biopsies were investigated.
(PDF)

S2 Table Primer Sequences. This table lists all sequences of the primers that where used in this investigation.
(XLSX)
