# Supplementary figures and images for: TLR-3 is Present in Human Adipocytes, but Its Signalling is Not Required for Obesity-Induced Inflammation in Adipose Tissue In Vivo
Source: PLoS One. 2015 Apr 13;10(4):e0123152. doi: 10.1371/journal.pone.0123152 (PMC4395029; doi:10.1371/journal.pone.0123152)

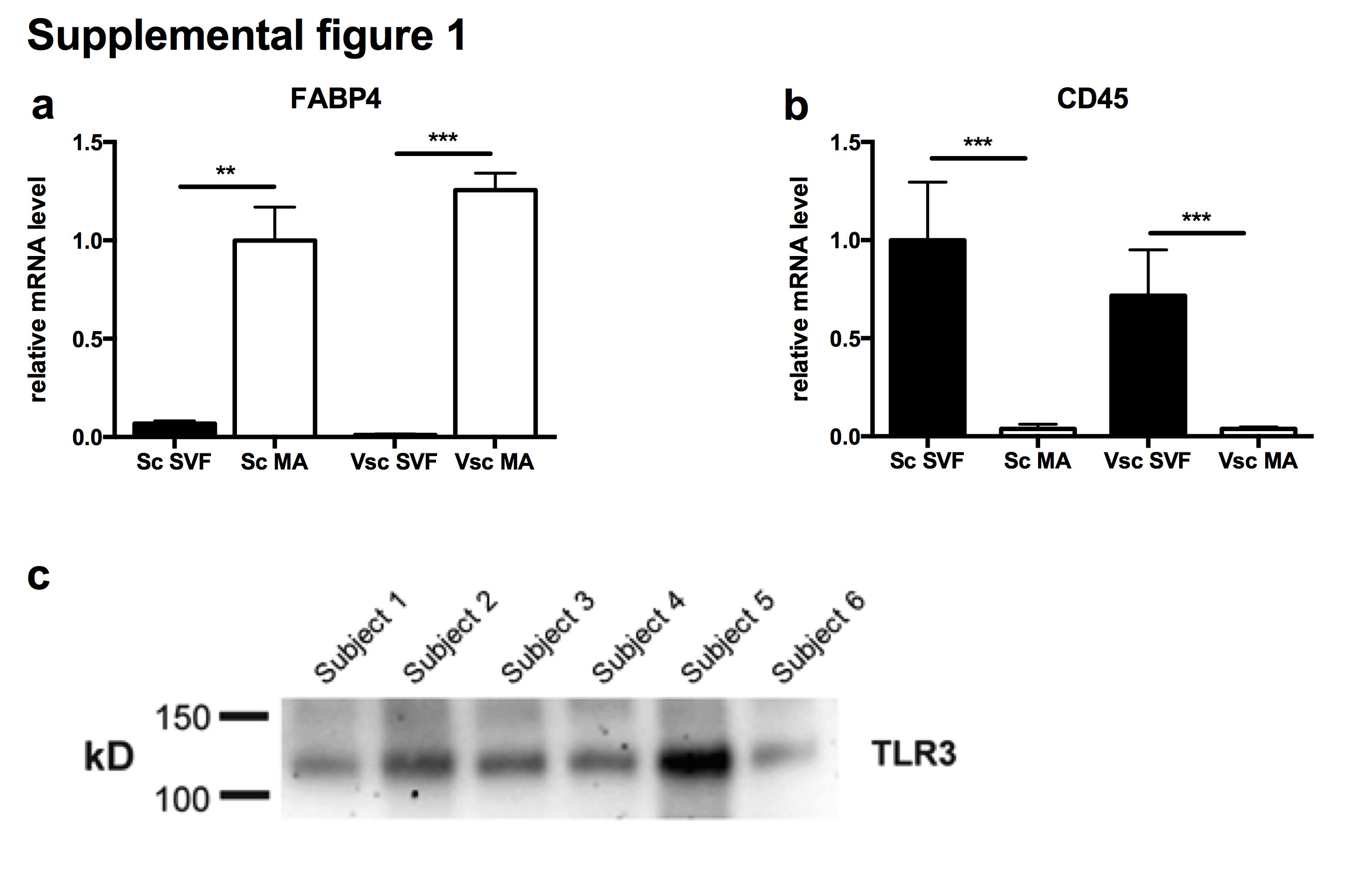

Supplement: S1 Fig — Biopsies from visceral- (VAT) and subcutaneous adipose tissue (SAT) were obtained from 4 healthy subjects and TLR expression was determined in stromal vascular fraction (SVF) and mature adipocytes (MA). mRNA levels of (a) FABP4 (b) CD45. (c) Western blot was used to confirm TLR-3 protein expression in human subcutaneous adipose tissue. * p<0.05, ** p<0.01, *** p<0.001. Data are shown as means ± SEM. (TIFF) [file pone.0123152.s001.tiff]

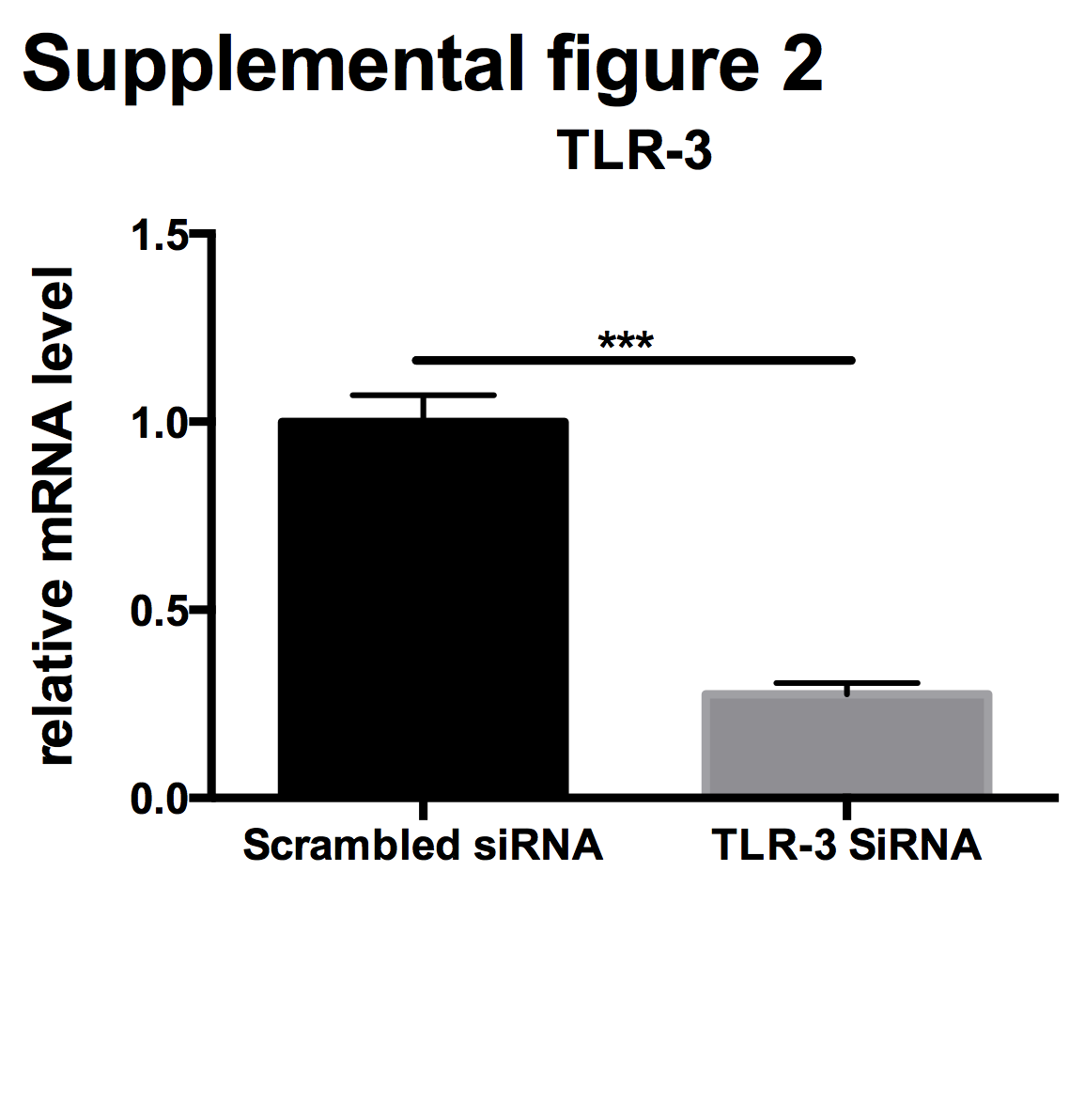

Supplement: S2 Fig — SGBS cells where transfected with small interference RNA against TLR-3 to reduce expression of TLR-3. Gene expression was determined after 72 hours. *** p<0.001. Data are shown as means ± SEM. (TIFF) [file pone.0123152.s002.tiff]

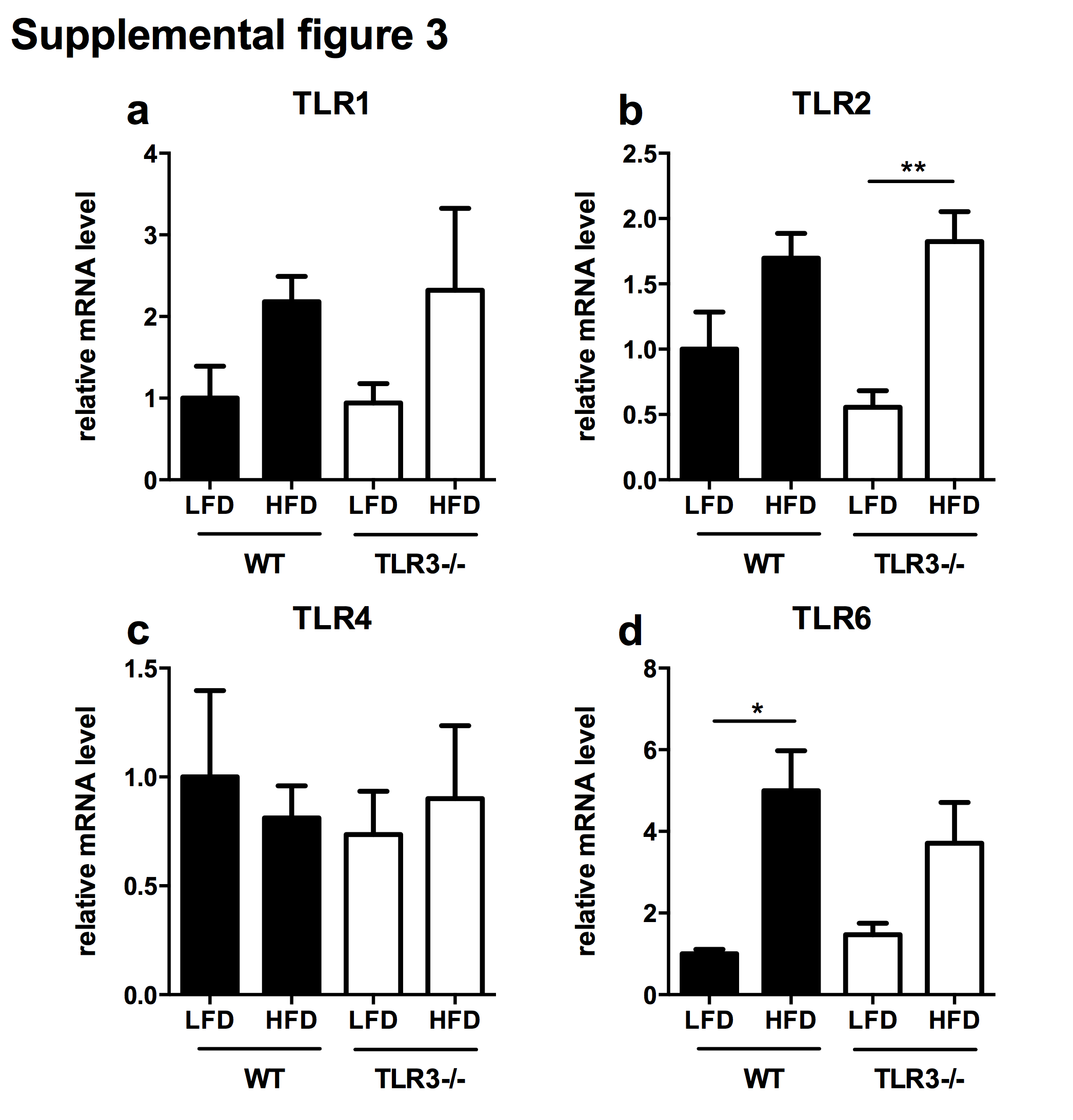

Supplement: S3 Fig — After 16 weeks of low fat diet (LFD) or high fat diet (HFD) intervention, adipose tissue of wild-type (WT) and TLR-3-/- mice was investigated for TLR expression. mRNA levels of (a) TLR-1, (b) TLR-2, (c) TLR-4 and (d) TLR-6 were measured. * p<0.05, ** p<0.01. Number of mice per group: WT-LFD n = 10; WT-HFD n = 10; TLR-3-/-LFD n = 7; TLR-3-/-HFD n = 9. Data are shown as means ± SEM. (TIFF) [file pone.0123152.s003.tiff]

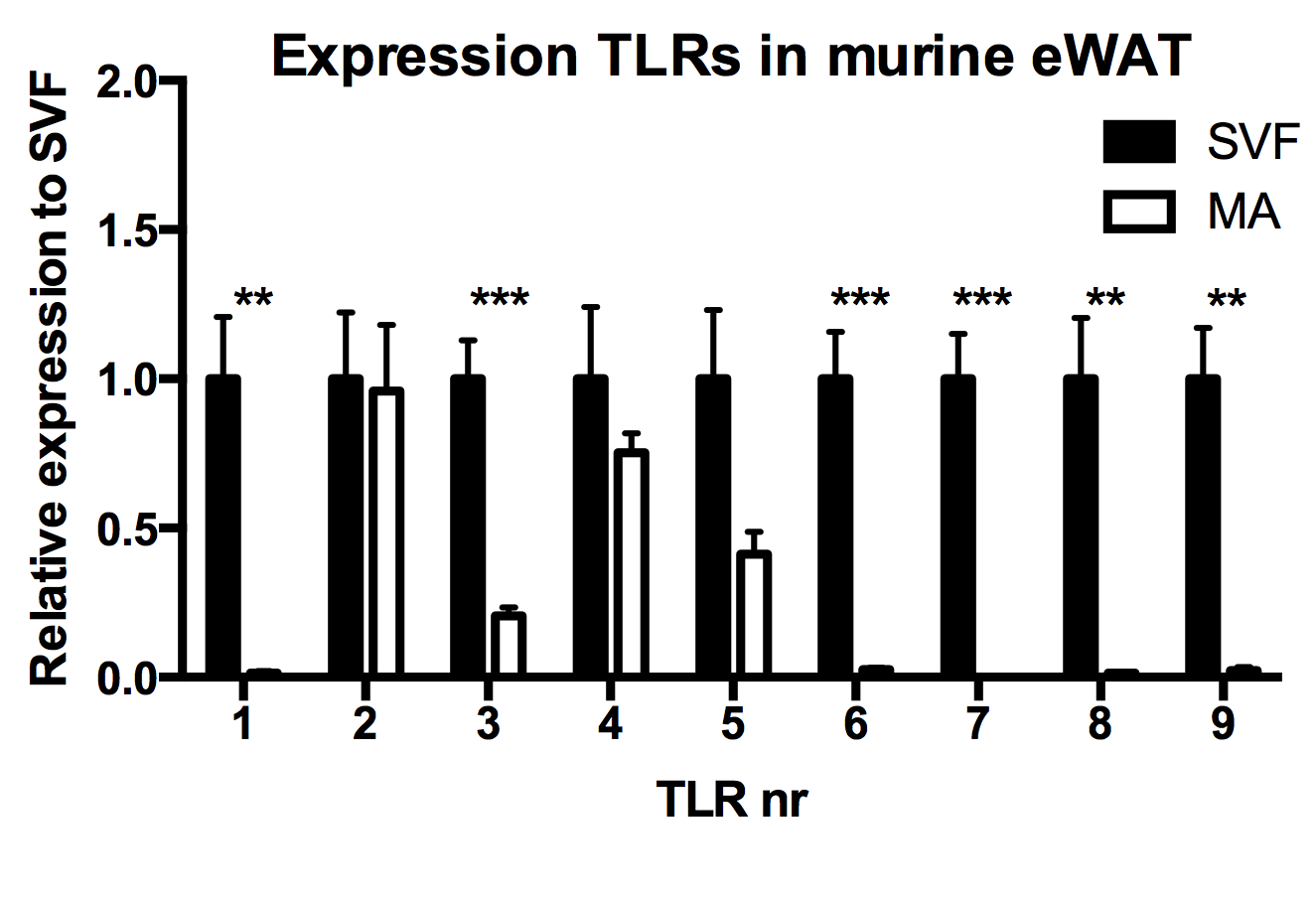

Supplement: S4 Fig — mRNA levels of 9 different TLRs were measured adipocytes versus stromal vascular cells in chow fed mice. Number of mice per group: adipocytes n = 9, stromal vascular cells n = 9. Data are shown as means ± SEM. * p<0.05, ** p<0.01, *** p<0.001. Data are shown as means ± SEM. (TIFF) [file pone.0123152.s004.tiff]
