# Supplementary material for: TLR-3 is Present in Human Adipocytes, but Its Signalling is Not Required for Obesity-Induced Inflammation in Adipose Tissue In Vivo
Source: PLoS One. 2015 Apr 13;10(4):e0123152. doi: 10.1371/journal.pone.0123152 (PMC4395029; doi:10.1371/journal.pone.0123152)
Supplement: S1 Table — This table lists information on the subjects of which adipose tissue biopsies were investigated. (PDF) [file pone.0123152.s005.pdf]

	Subcutaneous adipose tissue biopsies
N	80
% male	48%
Age (years) ± SD	54,7 ± 8,4
BMI (kg/m2) ± SD	27,7 ± 4,8
Fasting glucose (mm	5,1 ± 0,6
HOMA-IR ± SD	2,3 ± 2,1
hs-CRP (mg/L) ± SD	1,6 ± 1,7
